# Evaluation of the Savvy Caregiver Program for LGBTQIA Adults Living With Alzheimer's Disease and Related Dementias

**DOI:** 10.1093/geroni/igab046.1566

**Published:** 2021-12-17

**Authors:** Jason Flatt, Kiera Pollock, Yeonsu Song, Whitney Wharton, Joel Anderson

**Affiliations:** 1 University of Nevada Las Vegas, Las Vegas, Nevada, United States; 2 Los Angeles LGBT Center, Los Angeles, California, United States; 3 UCLA, Los Angeles, California, United States; 4 Emory University, Atlanta, Georgia, United States; 5 University of Tennessee at Knoxville, Knoxville, Tennessee, United States

## Abstract

Approximately 350,000 LGBTQIA+ older adults in the U.S. currently have Alzheimer’s disease and related dementias (ADRD), with projections nearing 1 million by 2030. LGBTQIA+ older adults face challenges in receiving adequate and inclusive care and caregiving support due to the inability to rely on traditional family networks, greater disability, and discrimination when seeking aging services. Working with the Los Angeles LGBT Center Aging in Community Initiative, we evaluated the: 1) Adaptation of the Savvy caregiver training program for care providers of LGBTQIA+ persons living with ADRD; and 2) Feasibility and acceptability of the program. Care providers were very satisfied with the program, strategies, information, and activities of the tailored Savvy program. For psychosocial outcomes, there were trends in greater care planning, increases in asking friends/family for support, and decreased loneliness. Additional research is needed on culturally-relevant aging services and behavioral interventions for care providers of LGBTQIA+ persons living with ADRD.

